# Bladder metastasis originating from gastric signet-ring cell carcinoma: a case report

**DOI:** 10.2144/fsoa-2023-0127

**Published:** 2024-05-20

**Authors:** Manel Moalla, Rania Hajji, Slim Charfi, Mona Boudabous, Hela Gdoura, Leila Mnif, Ali Amouri, Tahya Boudawara, Lassaad Chtourou, Nabil Tahri

**Affiliations:** 1 Gastroenterology department, Hedi Chaker Hospital, Sfax, Tunisia; 2 Pathology department, Habib bourguiba Hospital, Sfax, Tunisia

**Keywords:** adenocarcinoma, bladder metastasis, gastric cancer, signet-ring cell carcinoma

## Abstract

Bladder metastasis of gastric adenocarcinoma is a rare phenomenon. Hereby, we report a case of a 52-year-old patient who presented with upper gastro-intestinal bleeding and ascites and was diagnosed with gastric signet-ring cell carcinoma. A CT scan revealed peritoneal infiltration and anterior parietal thickening of bladder wall. Cystoscopy showed three budding lesions of the anterior wall of the bladder. He had an endoscopic resection. Histology concluded that the bladder was infiltrated by a poorly differentiated carcinoma with independent cells consistent with a gastric origin. The patient was to be treated with palliative chemotherapy.

## Plain language summary

We report a case of a 52-year-old patient who presented with symptoms such as stomach bleeding and a buildup of fluid in the stomach (ascites). The doctors diagnosed the patient with gastric cancer. The doctors also noted a thickening in the wall of the bladder during a CT scan. After more investigations, they discovered that the cancer from the stomach had spread to the bladder (metastasis).

## Abstract (plain language summary)

Bladder metastasis from gastric cancer is a rare situation.

Bladder adenocarcinoma should lead clinicians to search for a primary tumor.

Gastric cancer with bladder metastasis has poor outcome.

## Background

Bladder tumors rank first among urological cancers in Tunisia and second in the world after prostate cancer. Primary tumors of the bladder have transitional cell carcinoma in 95% at pathology exam. Adenocarcinomas constitute less than 1% of all bladder tumors and are usually due to metastatic lesions especially when it concerns signet-ring cell (SRC) carcinoma. The bladder is an unusual site of metastases (2%). When the histology shows SRC in the bladder, it is mandatory to look for a primary site, in particular stomach, colon and breast, before retaining bladder primitive tumor [1]. Hereby, we present a case of SRC carcinoma of the bladder originating from gastric cancer.

## Observation

A 52-year-old male patient, with no personal history of consumption of neither alcohol nor tobacco, with a family history of gastro-intestinal ulcer, presented with upper gastrointestinal bleeding 5 months ago. An upper endoscopy was performed at that time revealing an ulcerative stenosis of the duodenal bulb which was treated with proton pump inhibitors then concomitant quadruple therapy. The patient presented afterward with dysphagia and ascites with deterioration of his general well being consisting of body weight loss of about 6 kg, asthenia and anorexia. When hospitalized, he reported pollakiuria and some episodes of hematuria without fever.

The general examination showed a body mass index at 21 kg/m^2^, a general well being (OMS score = 0). Abdominal exam showed a moderate ascites without visceromegaly. Laboratory tests showed 6990 white blood cells/mcl, 14.6 g/dl of hemoglobin, thrombocytopenia at 110,000 platelets/mcl. The C reactive protein was negative. Liver and renal functions were normal. The analysis of the ascitic fluid was realized after performing percutaneous liquid puncture. It revealed a cell-rich exudate: 69 g/l of proteins and 350 white cells. The tumor markers blood test was positive for beta-HCG: 7.3 mUI/ml for an upper normal limit of 5 mUI/ml and negative for CA 19–9, CEA, PSA and AFP. Their dosage in the ascitic fluid showed very high levels of beta-HCG, CA 19–9 and CEA (458 mUI/ml, 1870 U/ml and 925 ng/ml, respectively).

**Figure 1.**
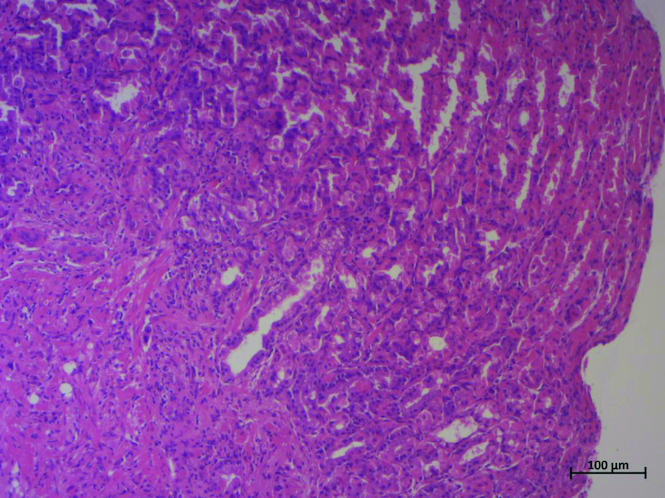
Poorly cohesive carcinoma invading the fundic mucosa (HE × 100).

**Figure 2.**
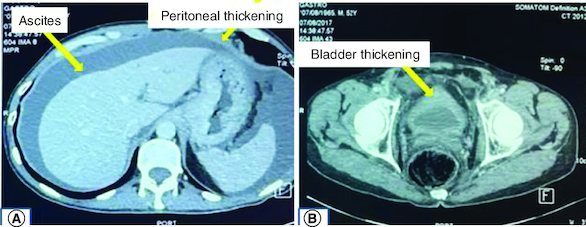
CT scan showing ascites, peritoneal and bladder parietal thickening. **(A)** Contrast enhanced CT scan demonstrating ascites and peritoneal thickening. **(B)** Contrast enhaced CT scan demonstrating bladder thickening.

Thus, we underwent a second upper endoscopy and colonoscopy. Upper endoscopy showed edematous and ecchymotic fundic gastropathy with healing of the bulbar ulcer. Histological examination revealed signet-ring cell adenocarcinoma of the stomach (Figure 1). The colonoscopy was normal. A CT scan revealed peritoneal and bladder parietal thickening associated with abundant ascites and multiple suspicious bone lesions (Figure 2).

**Figure 3.**
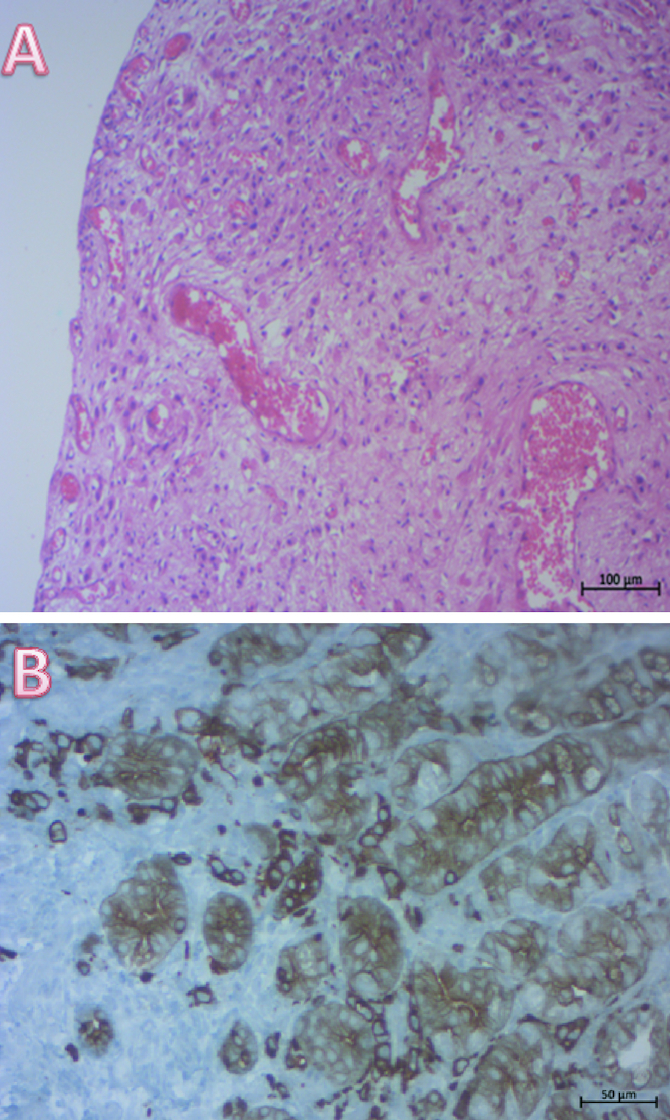
Histological examination of bladder biopsies. **(A)** Diffuse infiltration of the bladder mucosa by poorly cohesive neoplastic cells (HE × 100). **(B)** important immunostaining of neoplastic cells by keratin (×200).

The patient had a cystoscopy that showed three budding lesions at the anterior bladder wall that were biopsied. Histological exam showed a poorly differentiated carcinoma with SRC and immunohistochemical examination revealed CK7 +/ CK20+ profile with positive staining for the carcino-embryonic antigen (CEA) (Figure 3). Immunostaining was negative for CDX2. Thus, pathologists concluded to bladder infiltration of SRC gastric primary tumor.

The patient was diagnosed with SRC gastric adenocarcinoma with bladder metastasis, peritoneal carcinosis and probable bone metastases and he was referred to the oncology department for palliative care. He received zoledronic acid, oxaliplatin, capecitabin and epirubicin-based chemotherapy. He passed away 6 months after diagnosis.

## Discussion

Bladder cancer is ranked the second cancer in urological cancers after the prostatic tumors. As for bladder metastases, they are very uncommon representing less than 2% of bladder tumors.

Few tumors are known to metastasize to the bladder embracing two different ways: by contiguity which are colon (21%), prostate (19%), rectum (12%) and cervix cancer (11%) or by hematogenous or lymphatic path such as gastric cancer (4.3%), melanoma (3.9%), lung (2.8%) and breast cancer (2.5%) [2].

Including our case, there are 22 cases of gastric cancer spreading to the bladder [^1-5^]. The originality of our case is that the patient presented with dysphagia without any abnormality in the esophagus. Dysphagia may be related to pseudoachalasia or a tumoral infiltration of the cardia.

Bladder metastases are usually located in the trigone region or the bladder neck and are usually unique unlike primary bladder tumor [2]. It must be evoked especially when histology shows adenocarcnima which is the most encountered subtype in bladder metastases [5]. Signet-ring cell carcinoma should directly lead to the exploration of the digestive tract [6].

Urinary symptoms are found in approximately 20% of the cases of bladder metastases, consisting in macroscopic hematuria when the lesion is protuberant, which is the most common sign in bladder metastasis due to gastric cancer. In case of diffuse involvement of the bladder wall, irritative symptoms and hydronephrosis are predominant [1,7].

Immunochemistry may be helpful to distinguish between primary bladder tumor or metastasis. In gastric cancer, CK7 is usually positive and CK20 is negative. The opposite profile is consistent with colorectal origin. Positive expression rates of CK7 and CK20 in intestinal type of primary gastric cancer are 63% and 32%, respectively, whereas these rates are 75 and 42% in diffuse SRC type [1]. However, negative CDX2 which is frequently expressed in tumors of colorectal origin suggests rather primary bladder tumor. Its negativity in gastric cancer, is associated with bad outcome [8].

As for the treatment, patients with bladder metastasis of SRC gastric cancer are candidates for chemotherapy and radiotherapy which can slow down progression of the disease in some cases and may help control of urological symptoms [1]. The preferred regimens are combined chemotherapy drugs such as ECX (epirubicin, cisplatin and capecitabine), CX (cisplatin and capecitabine), EOX (epirubicin, oxaliplatin and capecitabine), IC (irinotecan and cisplatin). They have proven their superiority to the old regimen based on fluorouracil, doxorubicin and mitomycin [2,9]. Our patient received Zoledronic acid and EOX-based chemotherapy.

The overall outcome reported in the literature is very poor [2].

## Conclusion

Bladder metastasis from a gastric tumor is a very uncommon situation. Primary gastric tumor should be searched especially when the histological exam of bladder tumor shows adenocarcinoma. It has very bad outcome.

## References

[1] Okutur K, Eren OO, Demir G. Metastasis of Gastric Signet-Ring Cell Carcinoma to the Urinary Bladder: A Case Report and Review of the Literature. Case Rep. Oncol. Med. 2015, 1–6 (2015). 10.1155/2015/127516 PMC4539460 26346068

[2] Kazaz İO, Arslan A, Çolak F, Kazaz SN, Mungan S, Karagüzel E. Bladder metastasis of gastric signet-ring cell carcinoma. Urol. Case Rep. 22, 62–63 (2019). 30450283 10.1016/j.eucr.2018.10.013 PMC6234498

[3] Sato N, Kinoshita A, Imai N et al. A Case of Advanced Gastric Cancer with Bladder Metastasis. Gan To Kagaku Ryoho. 45(9), 1361–1363 (2018). 30237382

[4] Khoury R, Dragean, Annet L. Bladder Metastasis of Gastric Adenocarcinoma. J. Belg. Soc. Radiol. 103(1), 24 (2019). 30972381 10.5334/jbsr.1765 PMC6450255

[5] Nouioui MA, Saadi A, Chakroun M et al. Unusual Bladder Metastasis from a Primary Gastric Carcinoma: Two Case Reports and Review of Literature. Goel A, editor. Case Rep. Urol. 2020, 1–7 (2020). 10.1155/2020/8848841 PMC7683171 33274106

[6] Seddik Y, Jarroudi OA, Brahmi SA, Afqir S. Métastase vésicale d'un adénocarcinome gastrique en bague à chaton. Pan Afr. Med. J. 19, 206 (2019). 10.11604/pamj.2014.19.206.4966 PMC4369390 25821549

[7] Sharma PK, Vijay MK, Das RK, Chatterjee U. Secondary signet-ring cell adenocarcinoma of urinary bladder from a gastric primary. Urol. Ann. 3(2), 97–99 (2011). 21747602 10.4103/0974-7796.82177 PMC3130488

[8] Masood MA, Loya A, Yusuf MA. CDX2 as a prognostic marker in gastric cancer. Acta Gastro-Enterol. Belg. 79(2), 197–200 (2016). 27382937

[9] Kalra S, Manikandan R, Dorairajan LN, Badhe B. Synchronously detected secondary signet ring cell urinary bladder malignancy from the stomach masquerading as genitourinary tuberculosis. BMJ Case Rep. 2014, 206120 (2015). 10.1136/bcr-2014-206120 PMC4307070 25618874

